# Parentage test in broad-snouted caimans (*Caiman latirostris*, Crocodylidae) using microsatellite DNA

**DOI:** 10.1590/S1415-47572009005000077

**Published:** 2009-12-01

**Authors:** Rodrigo B. Zucoloto, Luciano M. Verdade, Priscilla M. S. Villela, Luciana C. A. Regitano, Luiz L. Coutinho

**Affiliations:** 1 Universidade Federal da Bahia, Salvador, BABrazil; 2 Laboratório de Ecologia Animal, Departamento de Ciências Biológicas, Escola Superior de Agricultura Luiz de Queiroz, Universidade de São Paulo, Piracicaba, SPBrazil; 3 Laboratório de Biotecnologia, Departamento de Zootecnia, Escola Superior de Agricultura Luiz de Queiroz, Universidade de São Paulo, Piracicaba, SPBrazil; 4 Embrapa Pecuária Sudeste, São Carlos, SPBrazil

**Keywords:** crocodilians, caiman, parentage, microsatellite DNA

## Abstract

In this study, microsatellite markers, developed for *Alligator mississipiensis* and *Caiman latirostris*, were used to assess parentage among individuals from the captive colony of *Caiman latirostris* at the University of São Paulo, in Piracicaba, São Paulo, Brazil. Many of the females in the colony were full siblings, which made maternal identification difficult due to genotypic similarity. Even so, the most likely mother could be identified unambiguously among offspring in most of the clutches studied. Two non-parental females displayed maternal behavior which would have misled managers in assigning maternity based on behavior alone. This set of variable loci demonstrates the utility of parentage testing in captive propagation programs.

*Caiman latirostris* is a medium-sized crocodilian that inhabits the wetlands and swamps of southeastern South America. The geographic distribution of the species covers the hydrographic basins of the Paraná and São Francisco Rivers, as well as a large number of small coastal drainage systems, from northeastern Brazil to northeastern Uruguay (Verdade, 1998; Verdade and Piña, 2006). The state of São Paulo, where this study was undertaken, is located in the center of the species range. *Caimanlatirostris* was considered an endangered species in Brazil from 1972 to 2003 (Vanzolini, 1972; Groombridge, 1982; Bernardes *et al.*, 1990; IBAMA 2003). The main causes for the decline in original populations were poaching for the leather trade, and habitat destruction, primarily for agricultural use (Brazaitis *et al.*, 1988; Verdade, 1997).

Since the late 1980's, the *Caiman latirostris* conservation program developed by the University of São Paulo (ESALQ, Piracicaba, São Paulo, Brazil) has been successful in breeding this species in captivity (Verdade and Sarkis, 1998; Verdade *et al.*, 2003). Due to the lack of information on nesting sites in the wild, and as this species is relatively common in Brazilian zoos, commercial farming operations have been proposed as the most feasible conservation strategy for the species in southern Brazil (Verdade, 1997, 2001).

Captive propagation efforts need to be guided by well-structured genetic management of the colony to prevent possible problems, such as founder effect, genetic drift and inbreeding depression (Ballou, 1992). Genetic management in the University of São Paulo captive colony is based on the establishment of a studbook in which individual pedigrees can be assessed and reproductive groups assembled, priority being given to nonrelated or least-related individuals (Verdade and Kassouf-Perina, 1993).

Molecular markers have been shown to be important tools in ecological and genetic research (Palo *et al.*, 1995; Verdade *et al.*, 2002). Microsatellites are among the best markers for parentage identification due to their high polymorphism (Craighead *et al.*, 1995; Garcia-Moreno *et al.*, 1996; Davis *et al.*, 2001a), so that with enough markers, overall exclusion probabilities of 99.8% can be obtained.

Microsatellite markers specifically developed for *Alligator mississipiensis* were tested with DNA from 21 species of the eight extant crocodilian genera (Glenn *et al.*, 1996, 1998). The tested primers were more efficient when amplifying orthologous loci in the DNA of species from the Alligatorinae subfamily than those from the Crocodylidae subfamily. However, amplification of *Caiman latirostris* DNA was not tested, and only one set of PCR conditions (the optimal conditions for *American alligators*) was used. Furthermore, the amount of intra-specific species polymorphism at the amplified loci was not determined for any of the other species. Therefore, it is possible that the use of different PCR conditions could permit amplification of additional loci from other species, especially *Caiman latirostris*. To date, there are 13 microsatellite markers specifically developed for *Caiman latirostris* (Zucoloto *et al.*, 2002). Since only some microsatellite markers can be used among closely related species (Moore *et al.*, 1991), we used microsatellite markers developed for *Caiman latirostris* and *Alligator mississipiensis* in the present study to assess parentage among individuals from the captive colony of *Caiman latirostris* at the University of São Paulo, in Piracicaba, São Paulo, Brazil.

The captive population sampled consisted of 16 adults and 24 hatchlings from the colony at the “Escola Superior de Agricultura - Luiz de Queiroz”, University of São Paulo, in Piracicaba, São Paulo, Brazil (Latitude: 22° 42.556' S, Longitude: 47° 38.246' W). Individuals were identified in the pens by tail-notch marking and interdigital tags, but are represented here by their Regional Studbook number (Verdade and Kassouf-Perina, 1993; Verdade and Andrade, 2003). Samples studied and identified by CL are maintained in the lysis buffer collection of the “Laboratório de Biotecnologia”, LPA, ESALQ, University of São Paulo, Piracicaba, São Paulo, Brazil. Alligatorinae. *Caiman latirostris*, Captive colony, ESALQ, University of São Paulo, Piracicaba, São Paulo, Brazil: 1-CL203, 2-CL25, 3-CL53, 4-CL106, 5-CL354, 6-CL355, 7-CL356, 8-CL357, 121-CL458, 123-CL460, 124-CL461, 125-CL462, 33-CL30, 34-CL10, 35-CL5, 36-CL13, 37-CL14, 38-CL70, 39-CL382, 40-CL383, 41-CL384, 42-CL385, 43-CL386, 63-CL434, 64-CL435, 65-CL436, 67-CL438, 142-CL479, 144-CL481, 146-CL483, 82-CL1, 83-CL9, 84-CL2, 85-CL3, 86-CL4, 87-CL19, 88-CL406, 92-CL410, 94-CL412, 96-CL414. Distribution of individuals in reproduction enclosures (ARN) was as follows: ARN1 (Father: 1-CL203, Possible mothers: 2-CL25, 3-CL53, 4-CL106, Clutch 1: 5-CL354, 6-CL355, 7-CL356, 8-CL357, Clutch 5: 121-CL458, 123-CL460, 124-CL461, 125-CL462); ARN3 (Father: 33-CL30, Possible mothers: 35-CL5, 34-CL10, 36-CL13, 37-CL14, 38-CL70, Clutch 2: 39-CL382, 40-CL383, 41-CL384, 42-CL385, 43-CL386, 8-CL357, Clutch 3: 63-CL434, 64-CL435, 65-CL436, 67-CL438, Clutch 6: 142-CL479, 144-CL481, 146-CL483) and ARN4 (Father: 82-CL1, Possible mothers: 84-CL2, 85-CL3, 86-CL4, 83-CL9, 87-CL19, Clutch 4: 88-CL406, 92-CL410, 94-CL412, 96-CL414). According to the Regional Studbook, the females 84-CL2, 85-CL3, 86-CL4, 35-CL5, 83-CL9, 34-CL10, 36-CL13, 37-CL14 and 87-CL19 are full sisters.

Animal immobilization was mechanical without anesthetics or muscle relaxants (Verdade, 1997). Blood was collected from the dorsal branch of the superior cava vein, which runs along the interior of the vertebral column of large reptiles (Olson *et al.*, 1975). After collection, blood was stored in a lysis buffer: 100 mM Tris-HCl, pH 8.0; 100 mM EDTA, pH 8.0; 10 mM NaCl; 0.5% SDS (w/v) as in Hoelzel (1992). DNA from these samples was purified by CTAB and chloroform extraction followed by isopropyl alcohol precipitation (Sambrook *et al.*, 1989).

Caimans build mound-nests, and females usually display parental behavior towards both the nest and hatchlings (Verdade, 1995; Thorbjarnarson, 1996). In this study, eggs were collected during the first 48 h after being laid and transferred to artificial incubators, (as described by Verdade *et al.*, 1992). Eggs and resulting hatchlings were identified by nest. Females guarding the nest were identified and assigned as possible clutch-mothers.

In the present study we used the markers *Ami*μ8, *Ami*μ13 and *Ami*μ20 developed for *Alligator mississipiensis* (Glenn *et al.*, 1998) and the markers *Cla*μ02, *Cla*μ05, *Cla*μ06, *Cla*μ07, *Cla*μ08, *Cla*μ09 and *Cla*μ10 developed for *C. latirostris* (Zucoloto *et al.*, 2002). Polymerase chain reaction (PCR) conditions were standardized for 25 μL with: 1 X specific buffer (Table 1, all buffers contain 300 mM Tris-HCl and 75 mM ammonium sulfate and differing concentrations of Mg^2+^ and pH), 0.2 mM each of dNTP, 0.4 μM of each primer pair, 0.2 U *Taq DNA polymerase*, and 100 ng DNA. The thermocycle program was: (1) 94 °C for 3 min, (2) 94 °C for 1 min, (3) primer specific annealing temperature for 1 min, (4) 72 °C for 1 min, (5) repeat steps 2, 3 and 4 for n cycles, (6) 72 °C for 7 min and (7) 4 °C until storage (Table 1). Products were stored at 4 °C until analyzing and scoring. PCR products were loaded into a Megabace 1000 DNA sequencer system for genotyping. Primers were labeled according to Table 1 and individuals genotyped by using the Genetic profiler program.

For logical reasons, such as the movement of individuals being restricted to individual enclosures, statistics were estimated by considering enclosures as though they were sampling units, as described above, ARN1 (N = 12) with one known parent (the father), three candidate parents (the possible mothers) and eight offspring from two clutches, ARN3 (N = 18) with one known parent (the father), five candidate parents (the possible mothers) and twelve offspring from three clutches, ARN4 (N = 10) with one known parent (the father), five candidate parents (the possible mothers) and four offspring from one clutch. The CERVUS 2.0 (Marshall *et al.*, 1998) program was used for calculating exclusion power and null allele frequencies for each locus (Table 2). The overall probability of exclusion for the maternity test by enclosure was computed with none parent known (Excl(1)) or with one parent known (Excl(2)) as shown in Table 2. CERVUS 2.0 was also used to assign maternity to possible mothers of offspring from the clutches in each enclosure, by employing the observed allele frequencies for enclosed populations to determine the statistical significance of the Δ value. This parameter was calculated by a simulation procedure that takes into account typing error rates and incomplete sampling for each possible mother, considering a given known father and offspring. At the end of this step, the possible mothers of each offspring were discriminated by Δ value and CI, *e.g.* the confidence interval, which could be either 80% or 95%, and corresponds to relaxed and restricted settings for CI, respectively, as shown in the last two columns of Table 3.

Exclusion power and null allele frequency estimates, for each locus and by enclosure, are presented in Table 2. The overall probability of exclusion for the maternity test, by enclosure and considering one parent known (Excl(2)), that is the case for this study, since the offsprings' father is always known as there was one single male by enclosure, was 99,1% for ARN1 (clutches 1 and 5), 96,4% for ARN3 (clutches 2, 3 and 6) and 96,3% for ARN4 (clutch 4).

According to the parentage test (Table 3) and on comparing genotypes (Table 4), the indicated mother for Clutch 1 is 4-CL106, in disagreement with the classification of female 2-CL25 as clutch-mother based solely on maternal behavior displayed by this individual and not the former. Nevertheless, the female 2-CL25 was excluded from maternity by six microsatellite markers, *Ami*μ*13*, *Cla*μ*02*, *Cla*μ*05*, *Cla*μ*06*, *Cla*μ*08* and *Cla*μ*10*, and the other possible mother, 3-CL53, by five microsatellite markers, *Ami*μ*13*, *Cla*μ*05*, *Cla*μ*06*, *Cla*μ*08* and *Cla*μ*10* (Table 4).

Female 34-CL10 was the behaviorally assigned mother of clutch 2 (Table 4). On the other hand, female 35-CL5 was assigned as the mother of 39-CL382 and 40-CL383 (Table 3), but she was excluded from maternity of the remaining hatchlings of clutch 2 by two microsatellite markers, *Cla*μ*02* and *Cla*μ*09* (Table 4). By the parentage test, female 36-CL13 was not assigned as mother, but could not be precluded from maternity of clutch 2 (Table 4). Female 37-CL14 was excluded from maternity of this clutch by microsatellite markers *Ami*μ*13* and *Cla*μ*09*, and female 38-CL70 was excluded from maternity of this clutch by *Ami*μ*13*, *Ami*μ*20*, *Cla*μ*02*, *Cla*μ*08* and *Cla*μ*09*. Maternity of clutch 2 remained uncertain for the females 34-CL10 and 36-CL13. Female 34-CL10 displayed parental behavior and was indicated as the mother by parentage testing of hatchlings 41-CL384, 42-CL385 and 43-CL386. In addition, she could not be definitely excluded as the mother of hatchlings 39-CL382 and 40-CL383. Female 36-CL13 could not be excluded from maternity of this clutch by any microsatellite marker (Table 4), although she was not indicated as the mother of any of the hatchlings by the parentage test. This suggests that female 34-CL10 is the actual mother of clutch 2, based on both behavioral and microsatellite evidence.

Female 35-CL5 was assigned as the actual mother of clutch 3, based on both behavioral and microsatellite evidence (Tables 3  and 4). The remaining females in enclosure ARN3 were excluded from maternity of clutch 3 by several microsatellite markers (Table 4): female 34-CL10 was excluded by *Cla*μ*08*, female 36-CL13 by *Cla*μ*08*, female 37-CL14 by *Ami*μ*13*, *Cla*μ*06* and *Cla*μ*08* and female 38-CL70 by *Ami*μ*13*, *Ami*μ*20*, *Cla*μ*06* and *Cla*μ*08*.

The behaviorally assigned mother of clutch 4, 83-CL9, was excluded from maternity of this clutch by microsatellite markers *Cla*μ*05*, *Cla*μ*06*, *Cla*μ*07* and *Cla*μ*08* (Table 4), whereas of the remaining females, 84-CL2 was excluded by *Cla*μ*08* and 86-CL4 by *Cla*μ*07* and *Cla*μ*08*. Female 85-CL3 could be neither excluded from maternity, nor indicated as the mother through parentage testing. Female 87-CL19 could not be excluded from maternity (Table 4), but was assigned as mother through parentage testing (Table 3). This was another case in which the molecularly assigned mother (87-CL19) was different from the behaviorally assigned (83-CL9).

Female 3-CL53 was distinguished as the mother of clutch 5 by both parentage microsatellite analysis (Table 3) as well as maternal behavior. The other two females in the same enclosure (ARN1) were excluded as mothers by microsatellite markers: female 2-CL25 by *Ami*μ*13, Cla*μ*02, Cla*μ*05, Cla*μ*06, Cla*μ*7* and *Cla*μ*08*, and female 4-CL106 by *Cla*μ*05, Cla*μ*06, Cla*μ*07* and *Cla*μ*08* (Table 4).

In clutch 6, female 35-CL5 was assigned as mother of 142-CL479 (Table 3), but was excluded from maternity of the remaining hatchlings by markers *Cla*μ*02, Cla*μ*07* and *Cla*μ*09* (Table 4). Female 36-CL13 was indicated as mother of 144-CL481 and 146-CL483 (Table 3), and could not be excluded from the remaining hatchlings by comparison among genotypes (Table 4). Female 34-CL10 was excluded as mother by *Cla*μ*07*, whereas female 37-CL14 was from maternity by *Ami*μ*13, Cla*μ*06* and *Cla*μ*09* and female 38-CL70 as mother by markers *Ami*μ*13, Ami*μ*20, Cla*μ*02, Cla*μ*06, Cla*μ*07, Cla*μ*08* and *Cla*μ*09* (Table 4). Based on the above, female 36-CL13 was assigned as mother of the clutch through microsatellite analysis, which was also in accordance with behavioral displays.

In four of the six clutches (2, 3, 5 and 6), mothers assigned by genetic analysis were in agreement with those indicated by maternal behavior: 34-CL10 for clutch 2, 35-CL5 for clutch 3 and 36-CL13 for clutch 6 (ARN3), 3-CL53 for clutch 5 (ARN1), see Tables 3  and 4.

For two of the six clutches (1 and 4), mothers assigned by genetic analysis were not the same as those indicated by maternal behavior. Behaviorally assigned mother for Clutch 1 (ARN1) was 2-CL25, whereas 4-CL106 was indicated as mother by microsatellite assay parentage test (Tables 3  and 4). In Clutch 4 (ARN4), female 83-CL9 displayed maternal behavior, whereas female 87-CL19 was indicated as mother by microsatellite assay parentage test (Tables 3  and 4).

With the set of markers used, it was possible to identify a single mother for all the offspring: clutches 1 (4-CL106), 2 (34-CL10), 3 (35-CL5), 4 (87-CL19), 5 (3-CL53) and 6 (36-CL13). Surprisingly, two of the females (33%) that displayed maternal behavior were not confirmed as actual mothers: 2-CL25 and 83-CL9. A display of maternal behavior by nonmothers can be explained as either a behavioral malfunction caused by the captive environment or species social adaptation as described in other vertebrates (Wrangham and Rubestein, 1986). Both hypotheses can be tested in future studies.

Farming operations are based on captive breeding and generally involve a small number of founders. Therefore, they require effective genetic management, in order to prevent genetic disorders as inbreeding depression (Foose, 1980). Assignment of mothers based exclusively on behavioral displays can lead to errors when assembling a Studbook and in establishing individual pedigrees. Under these circumstances microsatellite markers might be useful. In addition, these markers can also be useful in demographic and behavioral ecological studies in which the mating system and dispersal pattern are assessed based on parentage among individuals (*e.g.*, Verdade *et al.*, 2002).

## Figures and Tables

**Table 1:** Primer and amplification conditions.

Locus	Sequence 5'-3'	Buffer 10 X	Annealing ºC	Cycles	Label
*Ami*μ08a	CCTGGCCTAGATGTAACCTTC	A (7.5 mM MgCl2, pH 8.5)	55	30	FAM
*Ami*μ08b	AGGAGGAGTGTGTTATTTCTG				
*Ami*μ13a	CCATCCCCACCATGCCAAAGTC	A (7.5 mM MgCl2, pH 8.5)	64	35	FAM
*Ami*μ13b	GTCCTGCTGCTGCCTGTCACT				
*Ami*μ20a	TTTTTCTTCTTTCTCCATTCTA	F (10 mM MgCl2, pH 9.0)	58	30	TET
*Ami*μ20b	GATCCAGGAAGCTTAAATACAT				
*Cla*μ02a	CCTTCAGGACCCACTTTCTT	A (7.5 mM MgCl2, pH 8.5)	58	30	HEX
*Cla*μ02b	CGAATCCCTCTTCCCAAACT				
*Cla*μ05a	GCGTAGACAGATGCATGGAA	F (10 mM MgCl2, pH 9.0)	55	30	FAM
*Cla*μ05b	CAGTCTGAAGCTAGGGCAAA				
*Cla*μ06a	GAAATATGGGACAGGGAGGA	J (10 mM MgCl2, pH 9.5)	58	30	TET
*Cla*μ06b	GGTTGGCTGCATGTGTATGT				
*Cla*μ07a	CGGGGTCTTGGTGTTGACTA	F (10 mM MgCl2, pH 9.0)	58	30	TET
*Cla*μ07b	CGGGACCAGGAGCTGTATAA				
*Cla*μ08a	CAGCCACTGAAGGAATTGAC	F (10 mM MgCl2, pH 9.0)	55	30	FAM
*Cla*μ08b	CACATACCTGACCCAGCTTATC				
*Cla*μ09ª	ACAGGGGAAAAGAAGAGCTG	A (7.5 mM MgCl2, pH 8.5)	60	35	HEX
*Cla*μ09b	AAAATCCCCCACTCTTACCC				
*Cla*μ10a	TGGTCTTCTCTTCGTGTCCT	A (7.5 mM MgCl2, pH 8.5)	60	35	TET
*Cla*μ10b	ATGAGCCCCTCTATGTTCCT				

**Table 2:** Descriptive statistics by enclosure.

Locus	ARN1					ARN3					ARN4			
	N	Excl(1)	Excl(2)	Null		N	Excl(1)	Excl(2)	Null		N	Excl(1)	Excl(2)	Null
*Ami*μ08	12	0.099	0.173	-0.200		18	0.060	0.143	-0.124		10	0.000	0.000	+0.000
*Ami*μ13	9	0.272	0.439	+0.000		18	0.257	0.419	-0.166		10	0.262	0.431	-0.215
*Ami*μ20	12	0.042	0.143	-0.085		17	0.202	0.363	-0.122		10	0.016	0.082	-0.046
*Cla*μ02	12	0.123	0.253	-0.077		17	0.076	0.157	+0.376		10	0.171	0.309	-0.162
*Cla*μ05	12	0.428	0.607	-0.125		11	0.222	0.393	-0.150		10	0.192	0.360	+0.014
*Cla*μ06	12	0.217	0.382	-0.044		9	0.194	0.340	+0.000		7	0.146	0.258	+0.000
*Cla*μ07	12	0.162	0.304	-0.145		17	0.189	0.329	-0.113		10	0.125	0.188	+0.111
*Cla*μ08	12	0.391	0.569	-0.132		18	0.069	0.194	+0.033		9	0.309	0.481	+0.000
*Cla*μ09	12	0.215	0.363	-0.181		18	0.070	0.152	+0.385		6	0.162	0.304	+0.000
*Cla*μ10	12	0.199	0.368	-0.041		6	0.147	0.265	+0.000		10	0.128	0.258	-0.072
		0.921^a^	0.991^b^				0.806^a^	0.964^b^				0.816^a^	0.963^b^	

N - Individuals analyzed; Excl(1) - Exclusion power with no known parent; Excl(2) - Exclusion power with one known parent known; Null - Null allele frequency estimates; ^a^Total of exclusion power with no known parent; ^b^Total of exclusion power with one known parent.

**Table 3:** Parentage test results by enclosure and clutch.

	Offspring ID^a^	KP ID^b^	KP class	Offspring-KP loci compared^c^	Prob. non-exclusion	CP ID^d^	Offspring-CP loci compared^e^	Offspring-KP-CP loci compared^f^	LOD	Delta	CI
Clutch 1 (ARN1)	5 (10)	1 (10)	Typed	10 (0)	1.57E-03	4 (10)	10 (0)	10 (0)	4.20E+00	4.20E+00	*
	6 (10)	1 (10)	Typed	10 (0)	3.14E-04	4 (10)	10 (0)	10 (0)	6.38E+00	6.38E+00	*
	7 (10)	1 (10)	Typed	10 (0)	3.44E-03	4 (10)	10 (0)	10 (0)	3.77E+00	3.77E+00	*
	8 (10)	1 (10)	Typed	10 (0)	2.92E-03	4 (10)	10 (0)	10 (0)	4.14E+00	4.14E+00	*
Clutch 2 (ARN3)	39 (7)	33 (9)	Untyped	6 (0)	2.37E-01	35 (10)	7 (0)	6 (0)	1.93E+00	5.86E-01	+
	40 (7)	33 (9)	Untyped	6 (0)	2.19E-01	35 (10)	7 (0)	6 (0)	1.90E+00	6.79E-01	+
	41 (8)	33 (9)	Untyped	7 (0)	8.11E-02	34 (10)	8 (0)	7 (0)	2.76E+00	1.30E+00	+
	42 (7)	33 (9)	Untyped	6 (0)	5.52E-02	34 (10)	7 (0)	6 (0)	3.08E+00	6.53E-01	+
	43 (8)	33 (9)	Untyped	7 (0)	8.16E-02	34 (10)	8 (0)	7 (0)	2.93E+00	1.23E+00	+
Clutch 3 (ARN3)	63 (8)	33 (9)	Untyped	7 (0)	1.81E-01	35 (10)	8 (0)	7 (0)	2.57E+00	6.79E-01	+
	64 (7)	33 (9)	Untyped	6 (0)	2.05E-01	35 (10)	7 (0)	6 (0)	2.16E+00	6.79E-01	+
	65 (7)	33 (9)	Untyped	6 (0)	2.28E-01	35 (10)	7 (0)	6 (0)	2.08E+00	6.79E-01	+
	67 (8)	33 (9)	Untyped	7 (0)	3.63E-02	35 (10)	8 (0)	7 (0)	4.21E+00	3.49E+00	*
Clutch 4 (ARN4)	88 (9)	82 (10)	Typed	9 (0)	1.38E-01	87 (10)	9 (0)	9 (0)	2.72E+00	1.06E+00	*
	92 (8)	82 (10)	Typed	8 (0)	1.69E-01	87 (10)	8 (0)	8 (0)	2.28E+00	5.49E-01	+
	94 (7)	82 (10)	Typed	7 (0)	3.04E-01	87 (10)	7 (0)	7 (0)	2.07E+00	6.51E-01	+
	96 (8)	82 (10)	Typed	8 (0)	1.47E-01	87 (10)	8 (0)	8 (0)	2.37E+00	5.49E-01	+
Clutch 5 (ARN1)	121 (10)	1 (10)	Typed	10 (0)	1.65E-03	3 (10)	10 (0)	10 (0)	6.23E+00	6.23E+00	*
	123 (10)	1 (10)	Typed	9 (0)	8.27E-03	3 (10)	9 (0)	9 (0)	3.96E+00	3.96E+00	*
	124 (10)	1 (10)	Typed	9 (0)	5.72E-03	3 (10)	9 (0)	9 (0)	4.70E+00	4.70E+00	*
	125 (10)	1 (10)	Typed	9 (0)	1.71E-02	3 (10)	9 (0)	9 (0)	3.34E+00	3.34E+00	*
Clutch 6 (ARN3)	142 (8)	33 (9)	Untyped	7 (0)	1.87E-01	35 (10)	8 (0)	7 (0)	2.29E+00	6.91E-01	+
	144 (7)	33 (9)	Untyped	6 (0)	2.19E-01	36 (10)	7 (0)	6 (0)	5.92E-01	5.92E-01	+
	146 (8)	33 (9)	Untyped	7 (0)	3.29E-02	36 (10)	8 (0)	7 (0)	2.58E+00	2.09E+00	*

IDs in this table correspond to laboratory number. In the confidence interval column (CI) a + signal indicates that the result lies in the 80% confidence interval and an * signal indicates that the result lies on the 95% confidence interval.; ^a^(Offispring loci typed); ^b^(Known Parent loci typed); ^c^(Offspring-Known Parent loci mismatching); ^d^(Candidate Parent loci typed); ^e^(Offspring-Candidate Parent loci mismatching); ^f^(Offspring-Known Parent-Candidate Parent loci mismatching).

**Table 4:** Genotypes of *Caiman latirostris* individuals studied by clutch and enclosure.

Clutch 1 (ARN1) IDs	*Ami*μ08	*Ami*μ13	*Ami*μ20	*Cla*μ02	*Cla*μ05	*Cla*μ06	*Cla*μ07	*Cla*μ08	*Cla*μ09	*Cla*μ10
1-CL203 (Father)	115/115	264/270	126/152	203/205	165/211	227/227	181/215	111/117	161/165	218/222
2-CL25 (Behaviorally assigned mother)	115/117	*264/270*	126/154	*195/231*	*165/223*	*155/167*	181/215	*115/117*	165/165	*222/222*
3-CL53	115/117	*268/268*	126/126	203/203	*171/179*	*159/159*	215/249	*101/117*	165/177	*222/222*
4-CL106 (Microsatellite-assigned mother)	115/117	240/268	126/126	203/203	167/169	223/227	215/215	109/133	161/177	226/232
Clutch 1 mother alleles^1^	115 and 117	240 and 268	126	203	169	223 and 227	215	109 and 133	161 or 165 or 177	226 and 232
5-CL354	115/**115**	**268**/270	**126**/126	**203**/205	**169**/211	**223**/227	181/**215**	109/117	161/165	222/**226**
6-CL355	115/**117**	**240**/264	**126**/152	**203**/203	165/**169**	**223**/227	181/**215**	111/133	161/**177**	222/**232**
7-CL356	115/**117**	264/**268**	**126**/126	**203**/205	**169**/211	**227**/227	181/**215**	111/133	161/**177**	222/**232**
8-CL357	115/**117**	264/**268**	**126**/126	**203**/205	**169**/211	**227**/227	215/**215**	111/133	161/**177**	218/**226**

Clutch 2 (ARN3) IDs	*Ami*μ08	*Ami*μ13	*Ami*μ20	*Cla*μ02	*Cla*μ05	*Cla*μ06	*Cla*μ07	*Cla*μ08	*Cla*μ09	*Cla*μ10

33-CL30 (Father)	115/117	260/260	144/162	Undet^2^	157/169	223/223	181/203	111/125	161/165	226/226
34-CL10 (Microsatellite and behaviorally assigned mother)	115/115	254/272	124/124	195/203	197/197	227/227	215/215	125/125	161/165	226/226
35-CL5	115/115	254/272	124/124	*203/203*	197/197	227/227	215/215	109/125	*165/165*	224/226
36-CL13	115/115	254/272	124/124	195/203	195/197	227/227	203/215	125/125	161/165	222/222
37-CL14	115/115	*260/272*	124/124	195/203	195/197	239/239	203/215	125/125	*165/165*	222/222
38-CL70	115/115	*266/268*	*126/146*	*203/203*	195/197	239/239	215/215	*109/109*	*165/165*	226/226
Clutch 2 mother alleles^1^	115	254 and 272	124	195 and 203	197		215	125	161 and 165	
39-CL382	**115**/115	260/**272**	**124**/144	**203**/203	Undet^2^	Undet^2^	203/**215**	111/**125**	165/**165**	Undet^2^
40-CL383	**115**/117	**254**/260	**124**/144	**203**/203	Undet^2^	Undet^2^	203/**215**	**125**/125	165/**165**	Undet^2^
41-CL384	**115**/117	**254**/260	**124**/162	**203**/203	169/**197**	Undet^2^	181/**215**	**125**/125	161/**161**	Undet^2^
42-CL385	**115**/117	260/**272**	**124**/162	**195**/195	Undet^2^	Undet^2^	181/**215**	**125**/125	161/**161**	Undet^2^
43-CL386	**115**/115	**254**/260	**124**/144	**195**/195	157/**197**	Undet^2^	203/**215**	**125**/125	165/**165**	Undet^2^

Clutch 3 (ARN3) Ids	*Ami*μ08	*Ami*μ13	*Ami*μ20	*Cla*μ02	*Cla*μ05	*Cla*μ06	*Cla*μ07	*Cla*μ08	*Cla*μ09	*Cla*μ10

33-CL30 (Father)	115/117	260/260	144/162	Undet^2^	157/169	223/223	181/203	111/125	161/165	226/226
34-CL10	115/115	254/272	124/124	195/203	197/197	227/227	215/215	*125/125*	161/165	226/226
35-CL5 (Microsatellite and behaviorally assigned mother)	115/115	254/272	124/124	203/203	197/197	227/227	215/215	109/125	165/165	224/226
36-CL13	115/115	254/272	124/124	195/203	195/197	227/227	203/215	*125/125*	161/165	222/222
37-CL14	115/115	*260/272*	124/124	195/203	195/197	*239/239*	203/215	*125/125*	165/165	222/222
38-CL70	115/115	*266/268*	*126/146*	203/203	195/197	*239/239*	215/215	*109/109*	165/165	226/226
Clutch 3 mother alleles^1^	115	254 and 272	124	203	197	227	215	109 and 125	165	
63-CL434	**115**/115	260/**272**	**124**/162	**203**/203	169/**197**	Undet^2^	181/**215**	**125**/125	165/**165**	Undet^2^
64-CL435	**115**/115	**254**/260	Undet^2^	**203**/203	157/**197**	Undet^2^	203/**215**	**125**/125	165/**165**	Undet^2^
65-CL436	**115**/115	260/**272**	**124**/144	**203**/203	Undet^2^	223/**227**	Undet^2^	**125**/125	165/**165**	Undet^2^
67-CL438	**115**/117	**254**/260	**124**/162	**203**/203	Undet^2^	223/**227**	181/**215**	**109**/125	165/**165**	Undet^2^
82-CL1 (Father)	115/115	254/258	124/132	195/205	171/187	155/227	215/215	117/139	177/177	216/222
83-CL9 (Behaviorally assigned mother)	115/115	254/272	124/124	203/203	*165/179*	*227/227*	*215/215*	*101/117*	165/165	224/224
84-CL2	115/115	254/272	124/124	195/203	197/197	223/223	203/203	*109/109*	161/165	222/224
85-CL3	115/115	254/272	124/124	195/203	197/197	223/227	203/203	109/125	161/165	224/224
86-CL4	115/115	260/272	124/124	195/203	197/197	223/223	*215/215*	*125/125*	161/165	224/224
87-CL19 (Microsatellite-assigned mother)	115/115	260/272	124/124	203/203	197/197	223/223	203/203	109/125	165/165	224/224
Clutch 4 mother alleles^1^	115	272	124	203	197	223	203	109 and 125		224
88-CL406	115/**115**	254/**272**	**124**/124	**203**/205	171/**197**	**223**/227	**203**/215	117/**125**	Undet^2^	216/**224**
92-CL410	115/**115**	258/**272**	**124**/124	195/**203**	171/**197**	Undet^2^	**203**/215	117/**125**	Undet^2^	216/**224**
94-CL412	115/**115**	258/**272**	**124**/124	**203**/205	171/**197**	Undet^2^	**203**/215	Undet^2^	Undet^2^	216/**224**
96-CL414	115/**115**	258/**272**	**124**/132	195/**203**	187/**197**	Undet^2^	**203**/215	**109**/117	Undet^2^	216/**224**

Clutch 5 (ARN1) IDs	*Ami*μ08	*Ami*μ13	*Ami*μ20	*Cla*μ02	*Cla*μ05	*Cla*μ06	*Cla*μ07	*Cla*μ08	*Cla*μ09	*Cla*μ10

1-CL203 (Father)	115/115	264/270	126/152	203/205	165/211	227/227	181/215	111/117	161/165	218/222
2-CL25	115/117	*264/270*	126/154	*195/231*	*165/223*	*155/167*	*181/215*	*115/117*	165/165	222/222
3-CL53 (Microsatellite and behaviorally assigned mother)	115/117	268/268	126/126	203/203	171/179	159/159	215/249	101/117	165/177	222/222
4-CL106	115/117	240/268	126/126	203/203	*167/169*	*223/227*	*215/215*	*109/133*	161/177	*226/232*
Clutch 5 mother alleles^1^	115 and 117	268	126	203	171 and 179	159	215 and 249	101 and 117	161 or 165 or 177	222
121-CL458	115/**115**	264/**268**	**126**/126	**203**/203	**179**/211	**159**/227	181/**249**	**101**/117	161/**177**	218/**222**
123-CL460	115/**117**	Undet^2^	**126**/152	**203**/205	**171**/211	**159**/227	215/**215**	**101**/111	161/165	222/**222**
124-CL461	115/**117**	Undet^2^	**126**/126	**203**/205	**171**/211	**159**/227	215/**249**	111/**117**	161/**177**	222/**222**
125-CL462	115/**115**	Undet^2^	**126**/126	**203**/203	**179**/211	**159**/227	215/**249**	111/**117**	161/165	218/**222**

Clutch 6 (ARN3) Ids	*Ami*μ08	*Ami*μ13	*Ami*μ20	*Cla*μ02	*Cla*μ05	*Cla*μ06	*Cla*μ07	*Cla*μ08	*Cla*μ09	*Cla*μ10

33-CL30 (Father)	115/117	260/260	144/162	Undet^2^	157/169	223/223	181/203	111/125	161/165	226/226
34-CL10	115/115	254/272	124/124	195/203	197/197	227/227	*215/215*	125/125	161/165	226/226
35-CL5	115/115	254/272	124/124	*203/203*	197/197	227/227	*215/215*	109/125	*165/165*	224/226
36-CL13 (Microsatellite and behaviorally assigned mother)	115/115	254/272	124/124	195/203	195/197	227/227	203/215	125/125	161/165	222/222
37-CL14	115/115	*260/272*	124/124	195/203	195/197	*239/239*	203/215	125/125	*165/165*	222/222
38-CL70	115/115	*266/268*	*126/146*	*203/203*	195/197	*239/239*	*215/215*	*109/109*	*165/165*	226/226
Clutch 6 mother alleles^1^	115	254 and 272	124	195 and 203	197	227	203 and 215	125	161 and 165	
142-CL479	**115**/117	260/**272**	**124**/162	**203**/203	Undet^2^	223/**227**	203/**215**	111/**125**	165/**165**	Undet^2^
144-CL481	**115**/117	**254**/260	**124**/162	**203**/203	Undet^2^	Undet^2^	181/**203**	125/**125**	165/**165**	Undet^2^
146-CL483	**115**/117	260/**272**	**124**/162	**195**/195	169/**197**	Undet^2^	203/**203**	111/**125**	161/**161**	Undet^2^

^1^Mother alleles inferred from clutch-hatchling genotypes, ^2^Undetermined genotype: Father's alleles are underlined, Mother's alleles are in bold type, Excluded genotypes are in italics.
